# ACTH and PMX53 recover synaptic transcriptome alterations in a rat model of infantile spasms

**DOI:** 10.1038/s41598-018-24013-x

**Published:** 2018-04-10

**Authors:** Dumitru A. Iacobaş, Tamar Chachua, Sanda Iacobaş, Melissa J. Benson, Karin Borges, Jana Velíšková, Libor Velíšek

**Affiliations:** 1 Center for Computational Systems Biology, Prairie View AM University, Prairie View, TX 77446 USA; 2 D.P. Purpura Department of Neuroscience, Albert Einstein College of Medicine, New York, NY 10461 USA; 3 New York Medical College School of Medicine, Department of Cell Biology and Anatomy, Valhalla, NY 10595 USA; 4 New York Medical College School of Medicine, Department of Pathology, Valhalla, NY 10595 USA; 5 University of Queensland, School of Biomedical Sciences, Brisbane, Australia; 6 New York Medical College School of Medicine, Department of Neurology, Valhalla, NY 10595 USA; 7 New York Medical College School of Medicine, Department of Obstetrics and Gynecology, Valhalla, NY 10595 USA; 8 New York Medical College School of Medicine, Department of Pediatrics, Valhalla, NY 10595 USA

## Abstract

We profiled the gene expression in the hypothalamic arcuate nuclei (ARC) of 20 male and 20 female rats to determine the infantile spasms (IS) related transcriptomic alteration of neurotransmission and recovery following two treatments. Rats were prenatally exposed to betamethasone or saline followed by repeated postnatal subjection to NMDA-triggered IS. Rats with spasms were treated with ACTH, PMX53 or saline. Since ACTH, the first line treatment for IS, has inconsistent efficacy and potential harsh side effects, PMX53, a potent complement C5ar1 antagonist, was suggested as a therapeutic alternative given its effects in other epilepsy models. Novel measures that consider all genes and are not affected by arbitrary cut-offs were used, in addition to standard statistical tests, to quantify regulation and recovery of glutamatergic, GABAergic, cholinergic, dopaminergic and serotonergic pathways. Although IS alters expression of ~30% of the ARC genes in both sexes the transcriptomic effects are 3× more severe in males than their female counterparts, as indicated by the Weighted Pathway Regulation measure. Both treatments significantly restored the ARC neurotransmission transcriptome to the non-IS condition with PMX53 performing slightly better, as measured by the Pathway Restoration Efficiency, suggesting these treatments may reduce autistic traits often associated with IS.

## Introduction

Infantile spasms (IS; a term often interchangeably used with West syndrome) occur in 1:3200–1:3400 of live births. In 65% of cases, IS develops in response to brain damage^1^. West syndrome is characterized by clustered spasms during infancy (3–12 months of age, peaking around 6 months) that can involve flexors, extensors or both groups of muscles, interictal EEG hypsarrhythmia and mental regress^2,3^. Spasms occurring outside of the infancy period are termed epileptic spasms. Treatment of West syndrome is not only challenging, but also different from other seizure syndromes of childhood. Adrenocorticotropic hormone (ACTH) is the first line FDA-approved treatment. Other treatments include corticosteroids (prednisolone) and vigabatrin^4^. Unfortunately, the efficacy of these drugs is inconsistent and adverse effects are frequent and severe^5^. Often, successful elimination of spasms does not preclude development of mental decline^6^. Moreover, up to 35% of children with IS may develop traits of autism spectrum disorders (ASD)^7^, in contrast to the general population with approximately 1% prevalence of autism^8^.

Neuroinflammation, including that regulated via innate immune complement activation, represents a significant process involved in epilepsy^9–11^ and ASD^12^. Activation of the complement system via complement factor 5a through its receptor C5ar1 has been shown to drive pro-inflammatory processes in the brain^13^. When inhibited during status epilepticus (one of possible key initiating events in epileptogenesis), C5ar1 decreases production of tumor necrosis factor alpha (TNFα)^10^, which along with interleukin 1 beta (IL-1β), are key proinflammatory cytokines known to promote and initiate detrimental processes including microglial activation resulting in neuronal damage, and subsequent hyperexcitability^14^. PMX53 is a potent, selective, cyclic hexapeptide, C5ar1 antagonist, shown to have beneficial effects in acute seizure and chronic epilepsy models^10,15^. This C5ar1 antagonist is also neuroprotective^16^. Thus, by attenuating the inflammatory response PMX53 may compensate the alterations to excitatory/inhibitory balance in ASD^12,17^ which represents a common comorbidity of IS^7^.

We have developed a two-hit animal model of infantile spasms in rats, consisting of prenatal priming with corticosteroids and postnatal trigger of spasms with NMDA^18,19^. Prenatal priming with synthetic corticosteroids (or stress) increases susceptibility to postnatal NMDA trigger of flexion spasms and renders spasms responsive to ACTH treatment^20^. Spasms can be triggered by NMDA only during the period of brain development^21,22^ relevant for human IS^23^. Furthermore, EEG shows characteristics of hypsarrhythmia while ictal EEG displays electrodecremental response^24^ mimicking the human condition. The model has been independently reproduced and validated^25^.

Our initial study^18^ strongly indicated that the arcuate nucleus (ARC) was significantly involved in this model. We used timed c-fos imaging profiles (at 30, 60 and 120 min after trigger of spasms with NMDA) and already at 30 min we recorded significant increases in the ARC c-fos immunopositivity. This finding was further corroborated with [^14^C]2-deoxyglucose autoradiography which implied high glucose uptake, ergo high neuronal activity in the hypothalamic ARC.

In this study, we focused on the gene expression changes in ARC. As upstream of the hypothalamic paraventricular nucleus, ARC may affect release of endogenous CRF and via CRF it also controls endogenous ACTH release. We have previously reported that in prenatally betamethasone-exposed rats, prior to trigger of spasms, there are already modifications in the GABAergic and glutamatergic systems^26^. The rats demonstrate behavioral impairments reminiscent of ASD traits such as changes in ultrasonic vocalization (unpublished results).

**Figure 1:**
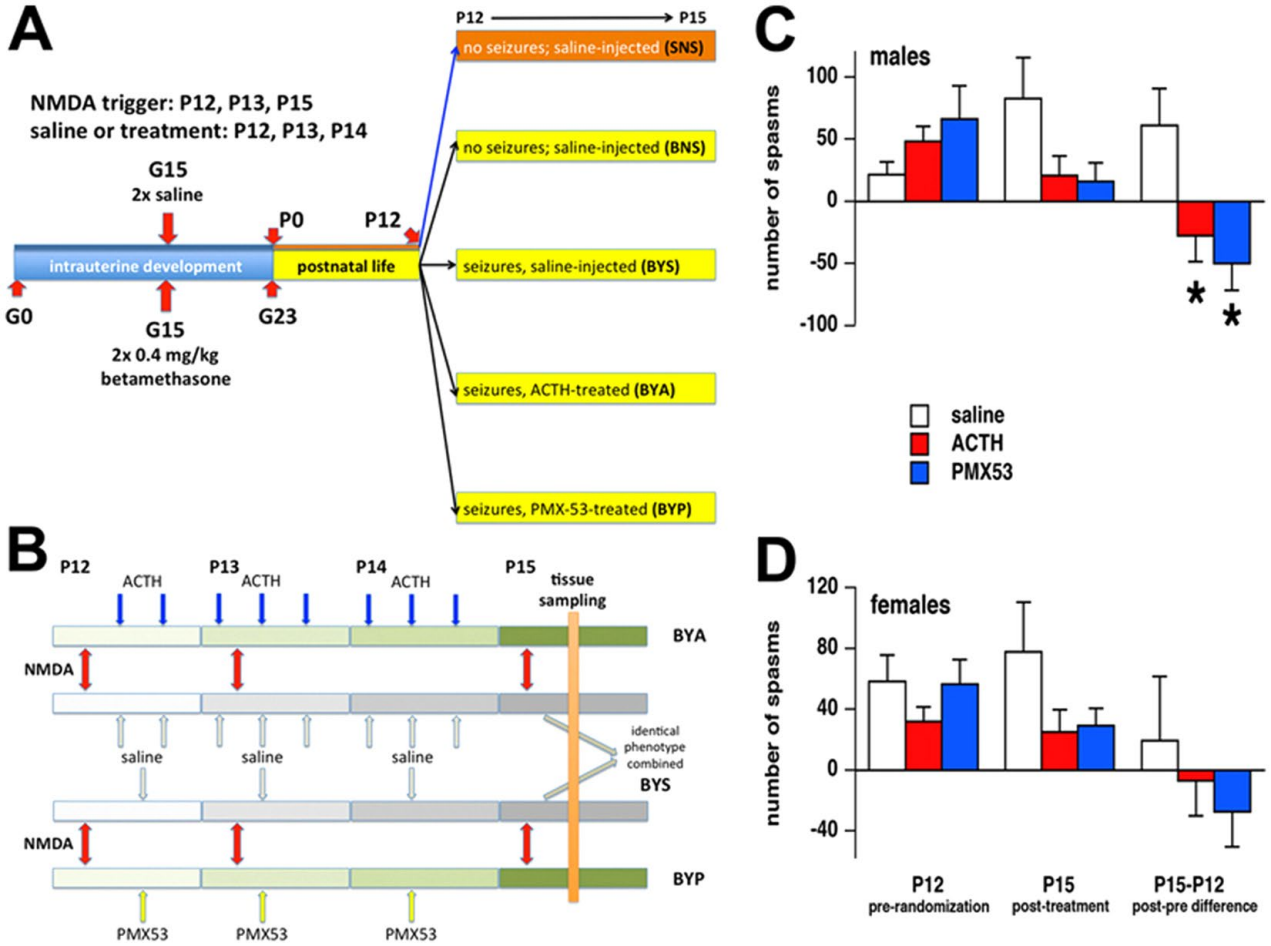
Experimental design and number of spasms before and after the treatments. (**A**) Groups of four rats used in this experiment. Pregnant females were injected either with two doses of betamethasone (0.4 mg/kg ip each) or saline on gestational day 15 (G15) and delivered on G23. On P12, a first round of spasms was triggered in some rats and then randomized into saline or ACTH or PMX53 groups (BYS, BYA, BYP). Some prenatally betamethasone-exposed rats received only saline instead of the NMDA trigger and saline instead of treatments (BNS). There was one additional group prenatally exposed to saline and postnatally also injected only with saline (SNS). (**B**) Treatment scheme of rats with triggered spasms. The red arrows identify NMDA triggers (morning of P12, P13 and P15). ACTH (each dose 0.3 mg/kg s.c) was administered at 14:00 and 21:00 on P12, at 07:00; 14:00 and 21:00 on P13 and P14. PMX53 (each dose 0.6 mg/kg s.c.) was administered at 14:00 on P12, P13 and P14. Control rats received saline with the same timing. (**C**,**D**) Pre and post treatment numbers of spasms in male **(C**) and female (**D)** rats. Left panels show efficacy of randomization (ANOVA F_(2,9)_ = 1.567; p = 0.260 for males and F_(2,9)_ = 1.010; p = 0.402 for females). Middle panels show number of spasms after completing the treatment on P15. While visually there were fewer spasms in ACTH or PMX53-treated rats, this difference was not significant in comparison to the saline-treated (ANOVA F_(2,9)_ = 2.649; p = 0.125 for males and F_(2,9)_ = 1.844; p = 0.213 for females). Right panels present the differences in the number of spasms in each individual rat after (P) and before (P12) the treatment. Treatment with both ACTH and PMX53 significantly decreased number of spasms between P12 and P15 if compared to the vehicle group for males (ANOVA F_(2,9)_ = 5.832; p = 0.024) but not for females (ANOVA F_(2,9)_ = 0.575; p = 0.582).

Here, we profiled the gene expression in the ARC of our model under several conditions following repeated spasms with and without ACTH or PMX53 treatments in primed animals (Fig. 1A,B). The purpose was to determine the extent by which the genomic fabrics of the synaptic neurotransmission change based on either prenatal betamethasone priming or occurrence of spasms, and how this change can resolve after relevant treatments. We also hypothesized that PMX53 would decrease occurrence of spasms and this effect would be similar to a standard drug used for IS treatment, ACTH, which is effective in our model of IS.

In previous publications^27–30^, we have developed the genomic fabric paradigm (GFP) to characterize the structured transcriptome associated to the most interconnected and stably expressed gene network responsible for a particular functional pathway (termed genomic fabric). Distinct from the mainstream quest for disease gene biomarkers, GFP looks for the global quantification of the alterations of the functional pathways in disease. We have also determined the transcriptomic effects of postnatal administration of glucorticosteroids^31^ and those related to the early-life seizure-induced neuroprotection^32^.

In this report, we have used GFP to quantify the remodeling of cholinergic (ACH), glutamatergic (GLU), GABAergic (GABA), dopaminergic (DA) and serotonergic (5HT) synaptic transmission pathways in the ARCs of betamethasone-primed NMDA-induced IS rats, and recovery following ACTH or PMX53 treatments. We have studied and compared five conditions; SNS, BNS, BYS, BYA and BYP. The three letter acronyms indicate whether the animal was prenatally subjected to betamethasone (B)/saline (treatment), had (Y)/had not (N) induced spasms and was postnatally treated with ACTH (A)/PMX53 (P) or just saline (S). For example, BYP represents betamethasone prenatal treatment, induced spasms and postnatal treatment with PMX53. Four males and four females were investigated in each condition.

## Results

### Effects of treatment with ACTH and PMX53 on spasms in male and female rats

Figure 1C,D show efficacy of randomization at P12 (before initiating the treatment, left panel), the fewer spasms in rats treated with ACTH or PMX53 (middle panel), and the difference for each individual rat after (P15) and before the treatment (P15-P12, right panel). Treatment with either ACTH or PMX53 decreased the number of spasms between P12 and P15 if compared to the vehicle group in both sexes. However, the decrease was statistically significant for males (ANOVA F_(2,9)_ = 5.832; p = 0.024) but not females (ANOVA F_(2,9)_ = 0.575; p = 0.582). The data clearly show that males appear to be more responsive to both treatments compared to females. Please note that only the animals entering microarray profiling are included here accounting for a small group size of n = 4 for each of the subgroups. We ran sample size analysis (G*Power) with our female data and determined that with the effect size of f = 0.7972 for a significant effect (α = 0.05) to occur, the subgroup size should be n = 7 [for power 1-β = 0.80].

### NMDA-induced spasms alter the neurotransmission transcriptome in a sex dependent manner

Detailed experimental procedure and raw and normalized gene expression data are publically available as GSE81061 and GSE84585 at http://www.ncbi.nlm.nih.gov/geo/.

**Figure 2:**
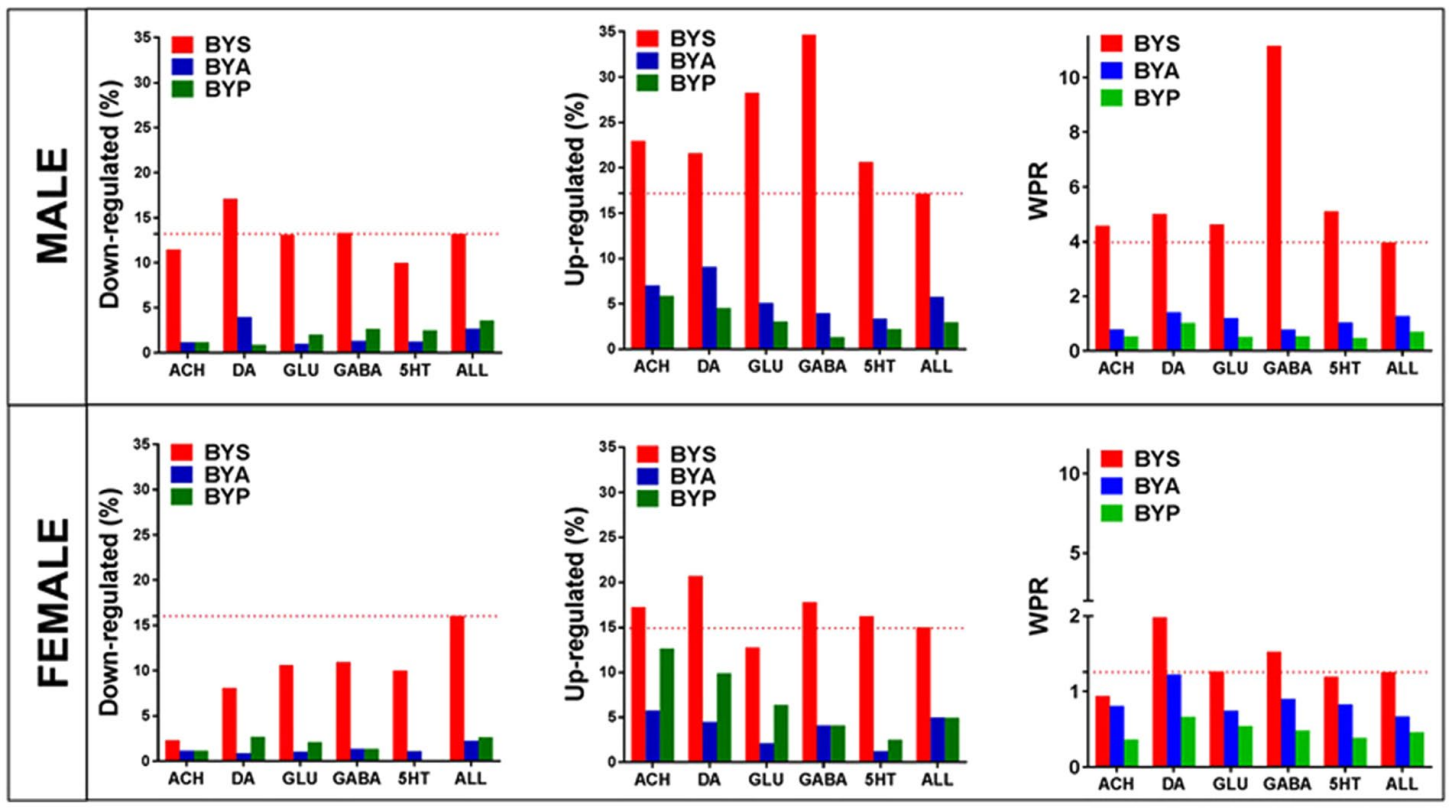
Transcriptomic alterations in response to NMDA-induced spasms in rats treated with ACTH (BYA), PMX53 (BYP) or just saline (BYS) compared to rats without IS (BNS). The alteration is presented as percentages of up-/down regulated genes and weighted pathway regulation (WPR) for the entire transcriptome (ALL) and each synapse pathway.

The induced spasms regulated large number of genes in both sexes: 30.37% of the 17,198 unigenes quantified in males and 30.92% of the 16379 unigenes quantified in females. As illustrated in Fig. 2, the transcriptomic alterations extended to all five neurotransmission types considered. Interestingly, although the overall percentage of significantly regulated genes was similar, the more accurate measure WPR (Weighted Pathway Regulation – see Methods) indicated females were significantly less affected than males (WPR_male_ = 3.966 vs WPR_female_ = 1.256). The largest sex difference was registered for GABA transmission, where WPR_male_ = 11.150 was 7.3 × higher than WPR_females_ = 1.529.

**Figure 3:**
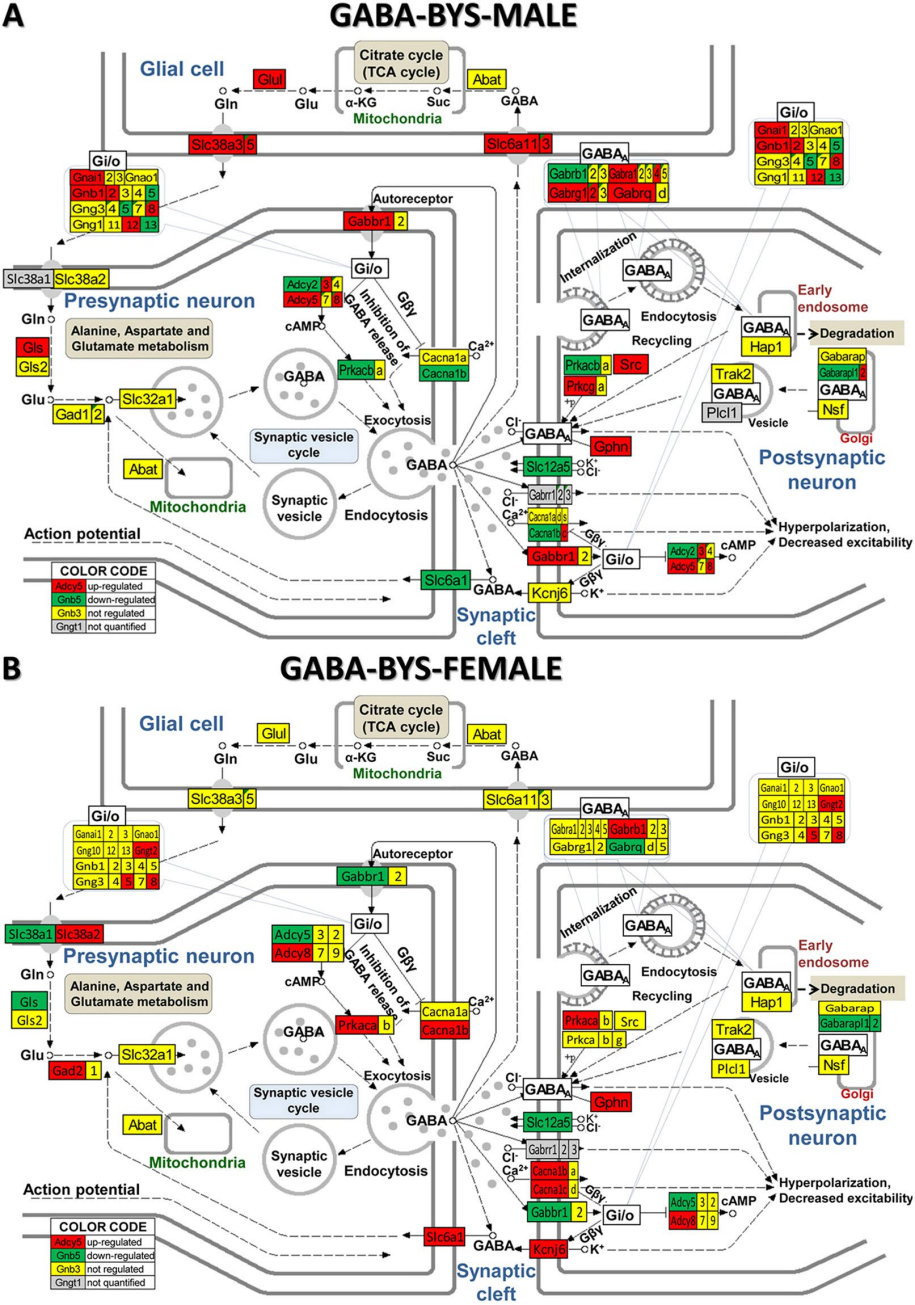
KEGG (www.kegg.jp/kegg/kegg1.html) map of regulation of the GABAergic synapse pathway in the arcuate nucleus of saline-treated betamethasone–primed male (**A**) and female (**B**) rats with NMDA-induced infantile spasms (BYS) compared to counterparts without spasms (BNS). Inset tables indicate how the composing genes of the labelled Gi/o (G-proteins) block are regulated in that condition. Gene symbol background indicates whether expression of a gene was upregulated (red), downregulated (green), unaltered (yellow) or not quantified (grey).

Moreover, as presented in Fig. 3 for the GABA synapse (and Supplementary Figs 1–4 for the other four synapses), spasms affected the genes in a very sex-dependent manner, with several genes being oppositely regulated in the two sexes. Thus, *Adcy5, Gabbr1, Gabarapl2, Gls* were up-regulated in males but down-regulated in females, while *Akt1, Cacna1b, Gng5, Plcb1, Slc6a1* were down-regulated in males but up-regulated in females. However, genes including: *Adcy3, Chrm4, Creb3l1, Fos, Fyn, Gani1, Gnb1, Glul, Jak2, Kcnj3, Kras, Nras, Slc18a3* were regulated in males but not in females, whilst: *Chrna4, Hras, Kcnj14, Kcnj6, Pik3ca, Prkaca* etc were regulated in females but not males.

Supplementary Table 1 lists the neurotransmission genes that were oppositely regulated by the IS in the two sexes.

### Recovery in response to ACTH and PMX53 treatments

The recovery was beyond any statistical doubt (heteroscedastic *t*-test) when comparing the percentages of regulated genes in the treated BYA and BYP conditions to the untreated BYS condition (Fig. 2). The p-values for significant change in percentage of down-regulated genes were: p-val_(ACTH)_ < 0.0002, p-val_(PMX53)_ <0.0011 for males and p-val_(ACTH)_ = 8.7E-6, p-val_(PMX53)_ = 2.0E-5 for females. The p-values for the changes in the up-regulated genes were: p-val_(ACTH)_ = 0.0022, p-val_(PMX53)_ = 5.8E-6 for males and p-val_(ACTH)_ = 0.0021, p-val_(PMX53)_ = 0.0021 for females.

**Figure 4:**
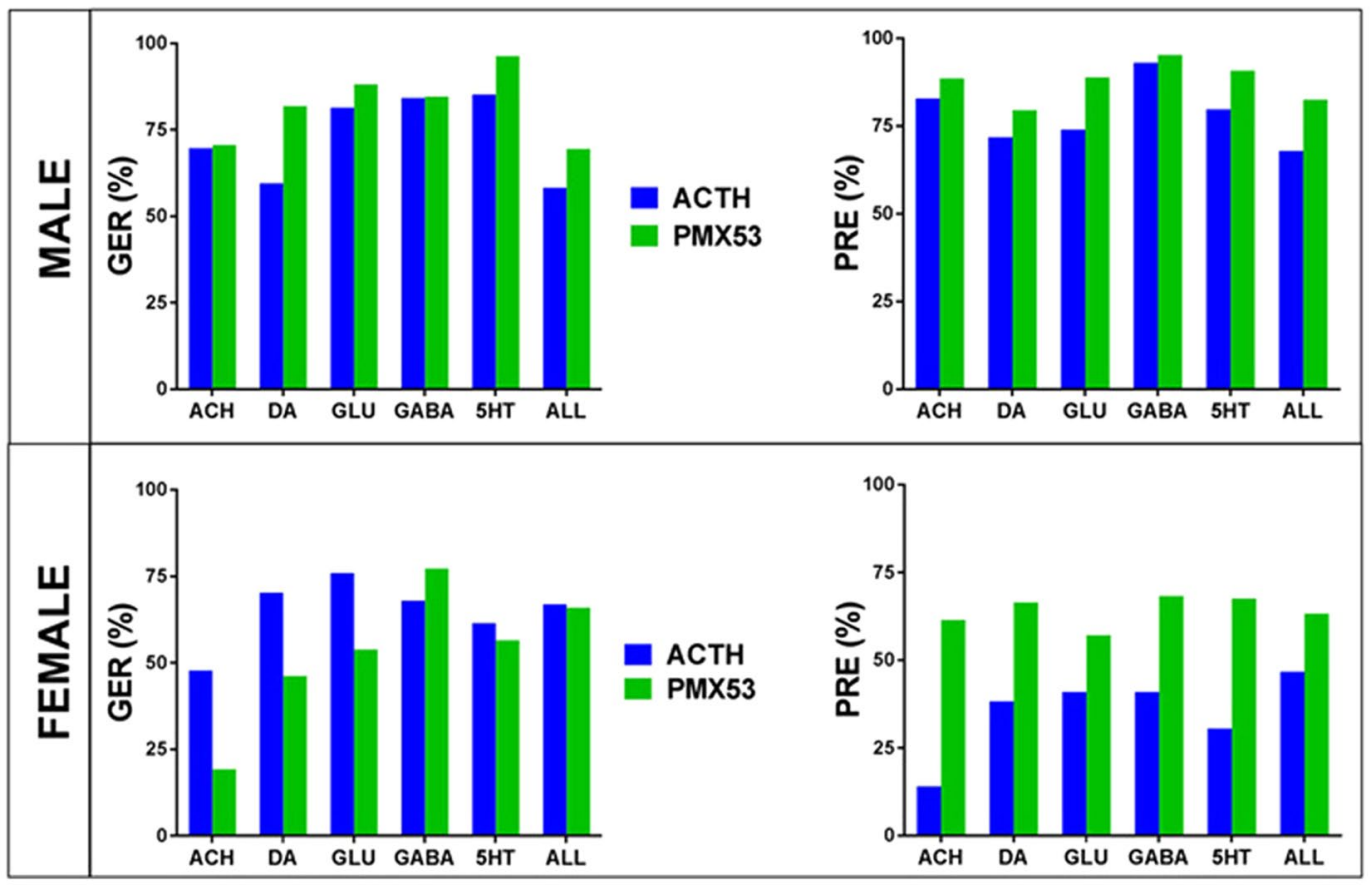
Recovery of gene expression following the two treatments in male and female rats with NMDA-induced spasms as quantified by the Gene Expression Recovery (GER) and Pathway Restoration Efficiency (PRE). Note that PRE indicates PMX53 is more efficient than ACTH for both sexes.

Figure 4 presents the recovery of the entire ARC transcriptome and of the transcriptomes associated to the five types of neurotransmission following either treatment using our original scores; GER (Gene Expression Recovery) and PRE (Pathway Restoration Efficiency).

GER (defined in Methods) was preferred over the traditional reduction of percentage of regulated genes because it considers both the IS-regulated genes restored by treatment and the genes not affected by IS but regulated by the treatment. Even more powerful than GER is PRE which takes into account alteration and recovery of all pathway genes, not only the significantly regulated, according to arbitrarily introduced criteria. Interestingly, according to PRE criterion PMX53 treatment appeared to be even more efficient than the classical ACTH treatment (p-val_male_ = 0.029, p-val_female_ = 0.001. However, the differences were not statistically significant with respect to the GER criterion (p-val_male_ = 0.106, p-val_female_ = 0.107). One may note that, while GER and PRE agrees for male that PMX53 was slightly more effective, for females they agree only for the GABA pathway, pointing to the errors caused by the arbitrary cut-offs in considering a gene as significantly regulated and neglecting the differential contributions of the genes to the global pathway transcriptomic alteration and recovery.

**Figure 5:**
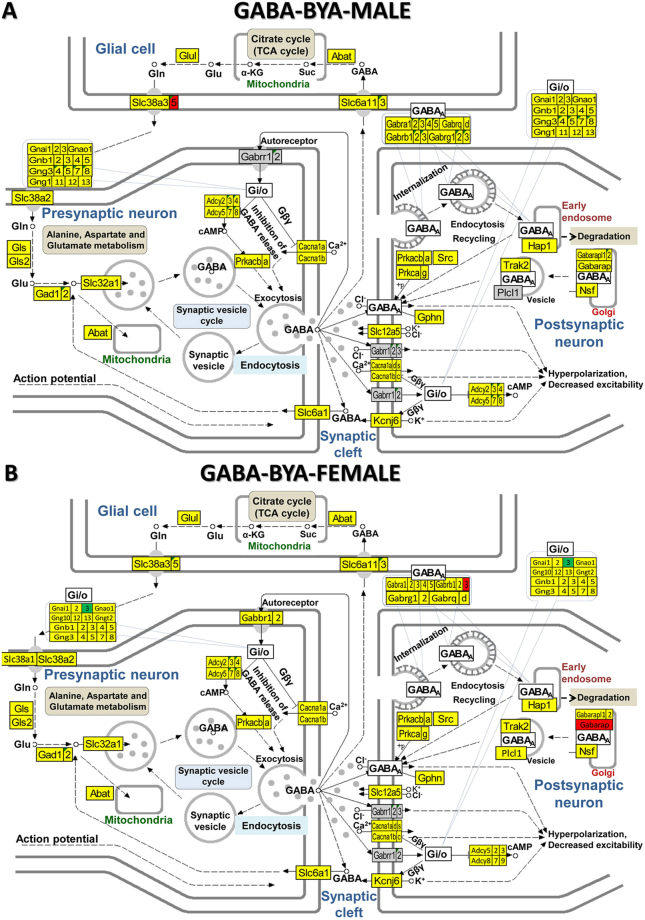
KEGG (www.kegg.jp/kegg/kegg1.html) map of regulation of the GABAergic synapse pathway in the arcuate nucleus of ACTH-treated betamethasone–primed male (**A**) and female (**B**) rats with NMDA-induced infantile spasms (BYA) compared to counterparts without spasms (BNS).

**Figure 6:**
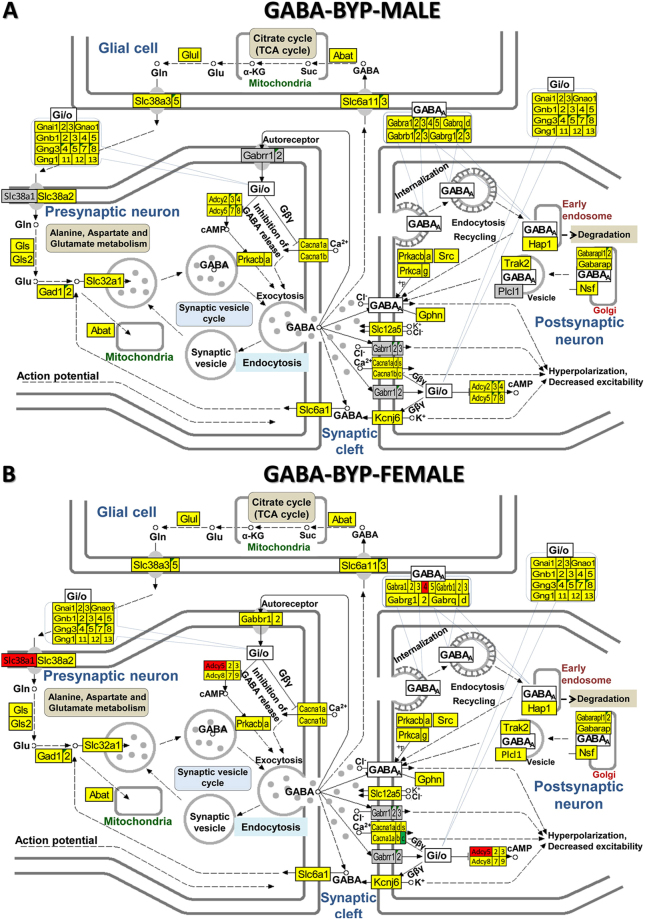
KEGG (www.kegg.jp/kegg/kegg1.html) map of regulation of the GABAergic synapse pathway in the arcuate nucleus of PMX53-treated betamethasone–primed male (**A**) and female (**B**) rats with NMDA-induced infantile spasms (BYA) compared to counterparts without spasms (BNS).

Figure 5 shows the GABAergic signalling pathway in male and female rats with NMDA-induced infantile spasms after ACTH treatment and Fig. 6 the GABAergic signalling pathway in male and female rats with NMDA-induced infantile spasms after PMX53 treatment. By comparing BYA (Fig. 5) and BYP (Fig. 6) conditions with the BYS condition (Fig. 3) the substantial recovery of the normal GABA transcriptome in both sexes resulting from either treatment is clear. There are also some differences between the results of the two treatments for the same sex as between sexes for the same treatment. We found similar recovery following the two treatments for both sexes in all other four synaptic pathways (illustrated in Supplementary Figs 5–12).

## Discussion

Numerous authors have identified genes altered in IS within glutamatergic and GABAergic neurotransmission^1,33–36^, indicated a role of serotonergic dorsal raphe nuclei in IS^37,38^ or suggested possible involvement of dopaminergic^39–41^ and cholinergic^42,43^ transmission in IS. Using gene expression microarrays and advanced analytical tools, our report, on the hypothalamic ARC of young male and female rats, provides additional findings related to IS-associated transcriptomic changes and recovery in response to two different treatments. It is the first part of a larger study involving both sexes and two well-defined brain regions: the ARC and the paraventricular node.

In a previous paper^26^, we presented how prenatal exposure to glucocorticoids affects the glutamatergic and GABAergic systems in the absence of spasms (compared BNS and SNS groups). Here, we studied what happens to betamethasone-primed rats after a series of NMDA-triggered spasms. Compared to the BNS group, BYS rats have had regulated all five neurotransmission pathways in percentages from 30% (5HT) to 48.0% (GABA) in males and from 20% (ACH) to 29% (DA, GABA) in females. Interestingly, while the average alteration of neurotransmission (39%) in males was larger than the average alteration of their transcriptome (30%), in females the opposite was true with neurotransmission genes (25% regulated) appearing more protected against IS effects than the overall transcriptome (31% regulated).

The standard analysis of the percentage of regulated genes is affected by the arbitrarily introduced cut-offs for the absolute fold-change (FC) and p-value of the *t*-test. We have replaced the fixed FC cut-off for all genes (e.g. 1.5×) with a value computed separately for each gene to account for the expression variability within biological replicas and technical noise of the probing spot(s) on the microarray. However, even with this refinement, the percentage analysis still ignores the non-significantly regulated genes, while considering the regulated ones as equal contributors to the pathway alteration. By contrast, the Weighted Pathway Regulation takes into account all pathway genes, weighting their contribution proportional to their normal expression level, net fold-change (FC-1) and confidence (1-p_val_) of their significant regulation.

WPR analysis confirmed that in males, the neurotransmission genes were more affected than the rest of the genome (WPR^neurotransmission^ = 6.090, WPR^all genes^ = 3.966), with GABA pathway topping the list of impaired synapses with WPR^GABA^ = 11.150. The value of WPR to characterize the pathways’ alterations is evident when comparing different synapses. Thus, while in males, the percentage of the regulated GABA genes exceeds by only 16% that of GLU genes, WPR^(GABA)^ score exceeds by 141% WPR^(GLU)^ because of the larger regulation of GABA than of GLU genes. WPR also indicated that in females the overall transcriptomic alteration was 3.2× less of the overall alterations in males and that the average neurotransmission (WPR^neurotransmission^ = 1.380) was just a little more affected than the entire transcriptome (WPR^all genes^ = 1.256).

Transcriptomic alterations do not stop at the expression level but also affect gene networks driving remodeling of entire synaptic transmission pathways. We proved recently^29^ that gene networks associated with glutamatergic transmission depend on species (mouse, rat), sex, brain region (hypothalamic arcuate nucleus, striatum and prefrontal cortex) and are affected by the prenatal exposure to betamethasone. We have shown also that neuropeptide Y coupling to glutamatergic and GABAergic transmission genes is strongly dependent on the sex hormones^44^. Transcriptomic networks (that may cross the cell’s boundaries)^45^ ensure the “transcriptomic stoichiometry” of the functional pathways so that the participating genes are expressed in the right proportions^46^.

We found that the IS-related transcriptomic alterations were largely corrected by both ACTH and PMX53 as indicated by reduced percentages of significantly regulated genes (Fig. 4). Thus, ACTH reduced significantly regulated ARC genes by 72% in males and by 77% in females, while PMX53 reduced the percentage by 78% in males and 75% in females. These percentages explain why spasms, which are the likely trigger for the observed gene expression changes, were blocked by both treatments. However, neither treatment was free of side effects, regulating genes that were not initially affected by IS. Therefore, instead of percent reduction of regulated genes we have used GER and PRE scores (defined in Methods). By considering the side effects, GER evaluates the recovery by ACTH in males at 58% and in females at 70%, compared to PMX53 at 69% in males and 66% in females. For genes listed in Supplementary Table 2, both treatments were “too” effective, switching the regulation from down- in BYS to up- in BYA or BYP, and vice-versa. We have also found genes (Supplementary Table 3) whose significant IS-related regulation was maintained by one treatment and reversed by the other.

Our previous studies indicated that ACTH suppresses the occurrence of NMDA-triggered spasms in both sexes^20,24^. Here we show that the complement C5ar1 receptor antagonist, PMX53, also reduced spasms, suggesting contribution of the innate inflammatory system, represented by C5a, to IS. The involvement of the inflammatory system in IS was recently documented by the increase of cytokines IL6 and IL17A in patients with IS prior to treatment initiation compared to healthy individuals^47,48^.

By enhancing the adrenal response with negative hypothalamic feedback of the hypothalamo-pituitary-adrenal axis ACTH may directly rectify some of the IS effects but the mechanisms of action of PMX53 are likely more complex. PMX53 has anticonvulsant effects in mouse models of epilepsy^10^, is neuroprotective^49^ and suppresses activation of the hypothalamo-pituitary-adrenal axis in an LPS-induced activation model^4,50^. These characteristics suggest that PMX53 may provide better protection against spasms and a better transcriptomic recovery from the IS phenotype.

In conclusion, treatment of the phenotype of spasms, either with a standard ACTH treatment or with a novel anti-inflammatory drug, PMX53, significantly rectified detrimental changes to gene transcription. Our microarray data indicate that the recovery of the normal level of gene transcription may correspond to the observed efficacy of the drug against the spasms.

## Methods

### Animals, seizures and treatments

We used offspring of timed-pregnant Sprague Dawley rats (Taconic Farms) purchased on gestational day 8 (G8). Rats were housed in our AAALAC-accredited animal facility for 7 days with free access to chow and water. All experiments were approved by New York Medical College Institutional Animal Care and Use Committee and conform to the Guide for the Care and Use of Laboratory Animals, 8th edition.

Experiments were performed using our model of infantile spasms^18,19^ consisting of prenatal priming and postnatal trigger of spasms. As illustrated in Fig. 1A, the rats were prenatally primed (on gestational/embryonic day 15; G15) with two doses of betamethasone (0.4 mg/kg each) delivered to pregnant mother intraperitoneally (controls received saline). After birth on G23 (postnatal day 0 = P0), rats were weighted and sexed. Later, rat pups received repeated administration of N-methyl-D-aspartic acid (NMDA) on P12, P13 and P15, which triggered spasms. After the spasms induced by the first trigger dissipated (on P12), the pups were randomized into treatment groups (Fig. 1B). Pups were treated with either ACTH (twice on P12 and three times a day on P13 and P14, each dose of 0.3 mg/kg sc; the effective treatment paradigm in our IS model)^20,24^, PMX53 (one daily dose of 0.6 mg/kg sc on P12, P13, P14 and P15; effective previously against seizure in epilepsy models)^10^ or saline as control, on P12, P13, P14 and P15. Spasms were followed for 60 min after the trigger and latency to onset of spasms from the trigger and number of spasms was calculated during the observation period. ANOVA with post-hoc Fisher’s Protected Least Square Degree test was used to evaluate data. Level of significance was preset to p < 0.05. For the microarray study, all rats were sacrificed at P15 under deep CO_2_ anaesthesia, the hypothalamic arcuate nuclei were dissected and immediately immersed in liquid nitrogen for later processing.

### Microarrays

We used our standard protocol^51^ for the extraction, purification, quantification, quality control, reverse transcription and fluorescent labelling of total RNA. RNA samples were hybridized with Agilent two-colour gene expression rat 4 × 44k arrays in the “multiple yellow” design that provides maximum flexibility in comparing the conditions and 100% usage of the resources^52^. Recently^53^, we have introduced a new combined criterion to consider a gene as differentially expressed between two conditions. The criterion requires that the absolute expression ratio exceeds the pooled contribution of the combined biological and technical variabilities in the compared conditions, and the p-value of the heteroscedastic (i.e. two-sample unequal variance) *t*-test of the means’ equality is below 0.05. The heteroscedastic *t*-test of the means’ equality was selected to account for the most likely case of two-tailed distributions of values with unequal variances.

#### Synapse Pathway regulation

Kyoto Encyclopedia of Genes and Genomes^54–56^ developed by Kanehisa Laboratories (http://www.genome.jp) was used to select the genes responsible for the rat cholinergic (ACH, map04725), glutamatergic (GLU, map04724), GABAergic (GABA, map04727), dopaminergic (DA, map04728) and serotonergic (5HT, map04726) transmissions. We quantified 97 distinct ACH genes in males and 87 in females, 120 DA genes in males and 112 in females, 110 GLU genes in males and 95 in females, 85 GABA genes in males and 73 in females, and 118 5HT genes in males and 80 in females. The lower number of distinct synapse genes quantified in females was caused by the higher biological variability among the female samples. Some of these genes were common to two or more synapses (e.g *Cacna1c* = calcium channel, voltage-dependent, L type, alpha 1 C subunit is common to all above-mentioned types of synaptic transmission). For each synapse, we identified and counted the genes that have been significantly up-/down-regulated in the animals with seizures and with or without treatment (BYS, BYA, BYP) with respect to the prenatally betamethasone exposed animals without seizures (BNS).

Weighted Pathway Regulation (WPR, Eq. 1)^57^ was used to better reflect the transcriptomic changes with synapse Γ (=ACH, GLU, GABA, DA, 5HT) pathway in condition α (=BYS, BYA, BYP) with respect to BNS.

1 $$\begin{array}{l}WP{R}_{{\rm{\Gamma }}}^{(\alpha )}=\mathop{\underbrace{{\langle {\mu }_{i}^{(BNS)}(|{x}_{i}^{(\alpha \to BNS)}|-1)(1-{p}_{i}^{(\alpha \to BNS)})\rangle }_{i\in {\rm{\Gamma }}}}}\limits_{{\rm{a}}{\rm{v}}{\rm{e}}{\rm{r}}{\rm{a}}{\rm{g}}{\rm{e}}\,{\rm{o}}{\rm{n}}\,{\rm{a}}{\rm{l}}{\rm{l}}\,{\rm{\Gamma }}-\text{pathway}\,{\rm{g}}{\rm{e}}{\rm{n}}{\rm{e}}{\rm{s}}},\quad {\rm{\forall }}\alpha =BYS,BYA,BYP\\ {\mu }_{i}^{(\beta )}={\rm{m}}{\rm{e}}{\rm{a}}{\rm{n}}\,\exp \,{\rm{l}}{\rm{e}}{\rm{v}}{\rm{e}}{\rm{l}}\,{\rm{o}}{\rm{f}}\,{\rm{g}}{\rm{e}}{\rm{n}}{\rm{e}}\, \mbox{``} {\rm{i}}\mbox{''}\,{\rm{i}}{\rm{n}}\,4\,{\rm{r}}{\rm{e}}{\rm{p}}{\rm{l}}{\rm{i}}{\rm{c}}{\rm{a}}{\rm{s}}\,{\rm{o}}{\rm{f}}\,{\rm{c}}{\rm{o}}{\rm{n}}{\rm{d}}{\rm{i}}{\rm{t}}{\rm{i}}{\rm{o}}{\rm{n}}\,\beta =\alpha ,BNS\\ {p}_{i}^{(\alpha \to BNS)}\equiv {p}_{val},\,\text{heteroscedastic}\,{\rm{t}} \mbox{-} \text{test}\,{\rm{o}}{\rm{f}}\,\text{means}\mbox{'}\,{\rm{e}}{\rm{q}}{\rm{u}}{\rm{a}}{\rm{l}}{\rm{i}}{\rm{t}}{\rm{y}}\\ {x}_{i}^{(\alpha \to BNS)}=\{\begin{array}{ccc}\frac{{\mu }_{i}^{(\alpha )}}{{\mu }_{i}^{(BNS)}} & if & {\mu }_{i}^{(\alpha )}\ge {\mu }_{i}^{(BNS)}\\ -\frac{{\mu }_{i}^{(BNS)}}{{\mu }_{i}^{(\alpha )}} & if & {\mu }_{i}^{(\alpha )} < {\mu }_{i}^{(BNS)}\end{array},\quad \text{fold} \mbox{-} \text{change}\,(\text{negative}\,{\rm{f}}{\rm{o}}{\rm{r}}\,\text{down} \mbox{-} \text{regulation})\end{array}$$

#### Transcriptomic recovery

We have evaluated the transcriptomic recovery by using both the Gene Expression Recovery (GER) and the Pathway Restoration Efficiency (PRE)^57^.

2 $$GE{R}_{{\rm{\Gamma }}}^{treat}=\frac{\{DX\}+\{UX\}-\{XD\}-\{XU\}}{\{DX\}+\{UX\}+\{XD\}+\{XU\}+\{DD\}+\{DU\}+\{UD\}+\{UU\}}\times 100 \% $$

where:*treat* = BYA, BYP,{*AB*} = number of genes that were *A*(*=U*(*up*)/*D*(*down*)/*X*(*not*) regulated) in theBYS/BNS comparison and *B*(*=D*/*U*/*X*) regulated in *treat/BNS* comparison.

GER subtracts the percentage of genes not affected by IS but regulated by the treatment (i.e. {XU}/all regulated genes, {XD}/all regulated genes) from the percentage of genes regulated by IS but restored by the treatment (i.e. {DX}/all regulated genes, {UX}/all regulated genes). Thus, GER takes into account both the positive effect of genes recovering their normal expression and the side effect of the treatment altering the genes otherwise not affected by the disease. We have previously used this measure (then termed TRE = Transcriptomic Recovery Efficiency) to evaluate the transcriptomic recovery efficacy of the bone marrow stem cell transplant in treating the chronic Chagasic cardiomyopathy^58^ and the myocardial infarct^59^. However, since for GER the contributions of all regulated/recovered genes are equal, we computed also PRE to determine the percent reduction of the WPR following the treatment.

3 $$PR{E}_{{\rm{\Gamma }}}^{(BYA)}=(1-\frac{WP{R}_{{\rm{\Gamma }}}^{(BYA)}}{WP{R}_{{\rm{\Gamma }}}^{(BYS)}})\times 100 \% ,\quad PR{E}_{{\rm{\Gamma }}}^{(BYP)}=(1-\frac{WP{R}_{{\rm{\Gamma }}}^{(BYP)}}{WP{R}_{{\rm{\Gamma }}}^{(BYS)}})\times 100 \% $$

Possible outcomes:

$$\begin{array}{l}WP{R}_{{\rm{\Gamma }}}^{(BYA/BYP)}=0\Rightarrow PR{E}_{{\rm{\Gamma }}}^{(BYA/BYP)}=100 \% ,\quad {\rm{full}}\,{\rm{recovery}}\\ WP{R}_{{\rm{\Gamma }}}^{(BYA/BYP)} < WP{R}_{{\rm{\Gamma }}}^{(BYS)}\Rightarrow 0 < PR{E}_{{\rm{\Gamma }}}^{(BYA/BYP)} < 100,\quad {\rm{positive}}\,{\rm{effect}}\\ WP{R}_{{\rm{\Gamma }}}^{(BYA/BYP)}=WP{R}_{{\rm{\Gamma }}}^{(BYS)}\Rightarrow PR{E}_{{\rm{\Gamma }}}^{(BYA/BYP)}=0,\quad {\rm{null}}\,{\rm{effect}}\\ WP{R}_{{\rm{\Gamma }}}^{(BYA/BYP)} > WP{R}_{{\rm{\Gamma }}}^{(BYS)}\Rightarrow PR{E}_{{\rm{\Gamma }}}^{(BYA/BYP)} < 0,\quad {\rm{negative}}\,{\rm{effect}}\end{array}$$

#### Gene networks

Genes of synaptic pathways were networked based on their coordinated (synergistic or antagonistic) expression^60^. Pearson product-momentum correlation coefficient between the (log_2_) expression levels of two genes within the four biological replicas of the same condition was used to determine whether the genes were (p < 0.05) synergistically/antagonistically/independently expressed. This analysis is based on the assumption that expressions of genes whose encoded proteins are functionally linked should be coordinated for their full use in the pathway.

## Electronic supplementary material

Supplementary Material
