# Supplementary material for: ACTH and PMX53 recover synaptic transcriptome alterations in a rat model of infantile spasms
Source: Sci Rep. 2018 Apr 10;8:5722. doi: 10.1038/s41598-018-24013-x (PMC5893534; doi:10.1038/s41598-018-24013-x)
Supplement: Supplementary file 1 — Supplementary Material [file 41598_2018_24013_MOESM1_ESM.docx]

**SUPPLEMENTARY MATERIAL**

**ACTH and PMX53 recover synaptic transcriptome alterations in a rat model of infantile spasms**

Dumitru A Iacobas, Tamar Chachua, Sanda Iacobas, Melissa J. Benson, Karin Borges, Jana Velíšková and Libor Velíšek

**Supplementary Table S1**

**Neurotransmission genes oppositely regulated by IS in the two sexes.** Average gene expression levels in BYS condition were compared to those in the BNS condition. Numbers in BYS columns represent the fold-change (negative for down-regulation). CUT is the absolute fold-change cut-off determined for each gene separately to exceed the combined contribution of the technical noise of the probing spot(s) and the expression variability within biological replicas in the two conditions (see Methods). Note that the quality of the biological material and the hybridization allowed for many genes lower that standard 1.5x fold-change cut-off. p is the p-value of the heteroscedastic *t*-test of the equality of the average expression levels in the compared conditions. Grey background indicates down-regulation.

**Supplementary Table S2**

**Genes whose significant up-/down-regulation in the BYS condition shifted to opposite regulation by both treatments (BYA and BYP conditions).** Average gene expression levels in BYS, BYA and BYP conditions were compared to those in the BNS condition. Numbers in columns labeled as BYS, BYA and BYP represent the fold-change (negative for down-regulation). CUT is the absolute fold-change cut-off determined for each gene separately for every comparison (e.g. BYS vs BNS) to exceed the combined contribution of the technical noise of the probing spot(s) and the expression variability within biological replicas. Grey background indicates down-regulation.

**Supplementary Table S3**

**Genes whose significant up-/down-regulation in the BYS condition were maintained by one treatment and flipped by the other.** Average gene expression levels in BYS, BYA and BYP conditions were compared to those in the BNS condition. Numbers in columns labeled as BYS, BYA and BYP represent the fold-change (negative for down-regulation). CUT is the absolute fold-change cut-off determined for each gene separately for every comparison to exceed the combined contribution of the technical noise of the probing spot(s) and the expression variability within biological replicas. Grey background indicates down-regulation.

**SUPPLEMENTARY FIGURES**

**
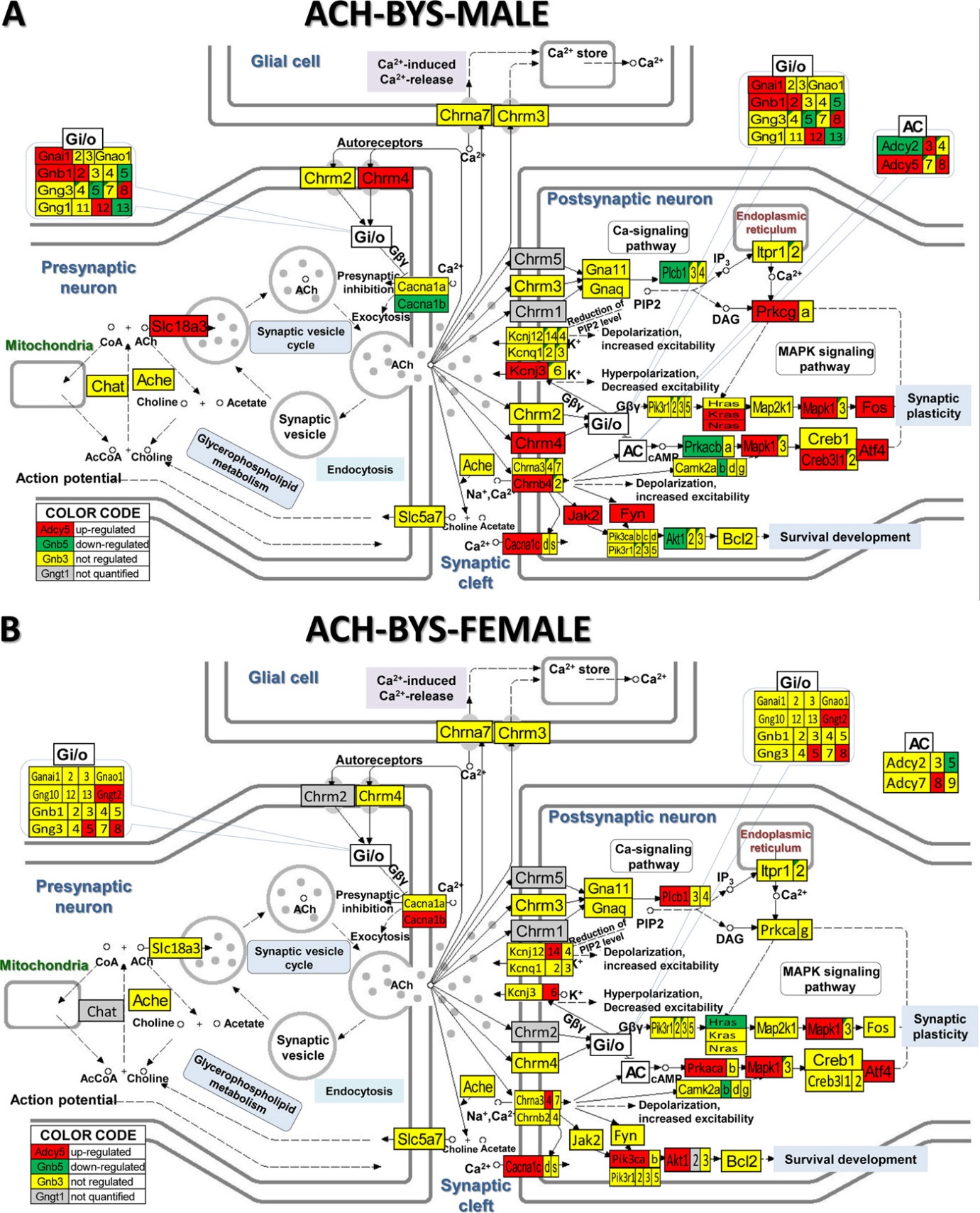
Supplementary Figure S1**

**KEGG (**www.kegg.jp/kegg/kegg1.html**) map of regulation of the cholinergic synapse pathway in the arcuate nucleus of saline-treated betamethasone–primed male (A) and female (B) rats with NMDA-induced infantile spasms (BYS) compared to counterparts without spasms (BNS).** Inset tables indicate how the composing genes of the labelled Gi/o (G-proteins) block are regulated in that condition.

**Supplementary Figure S2**

**
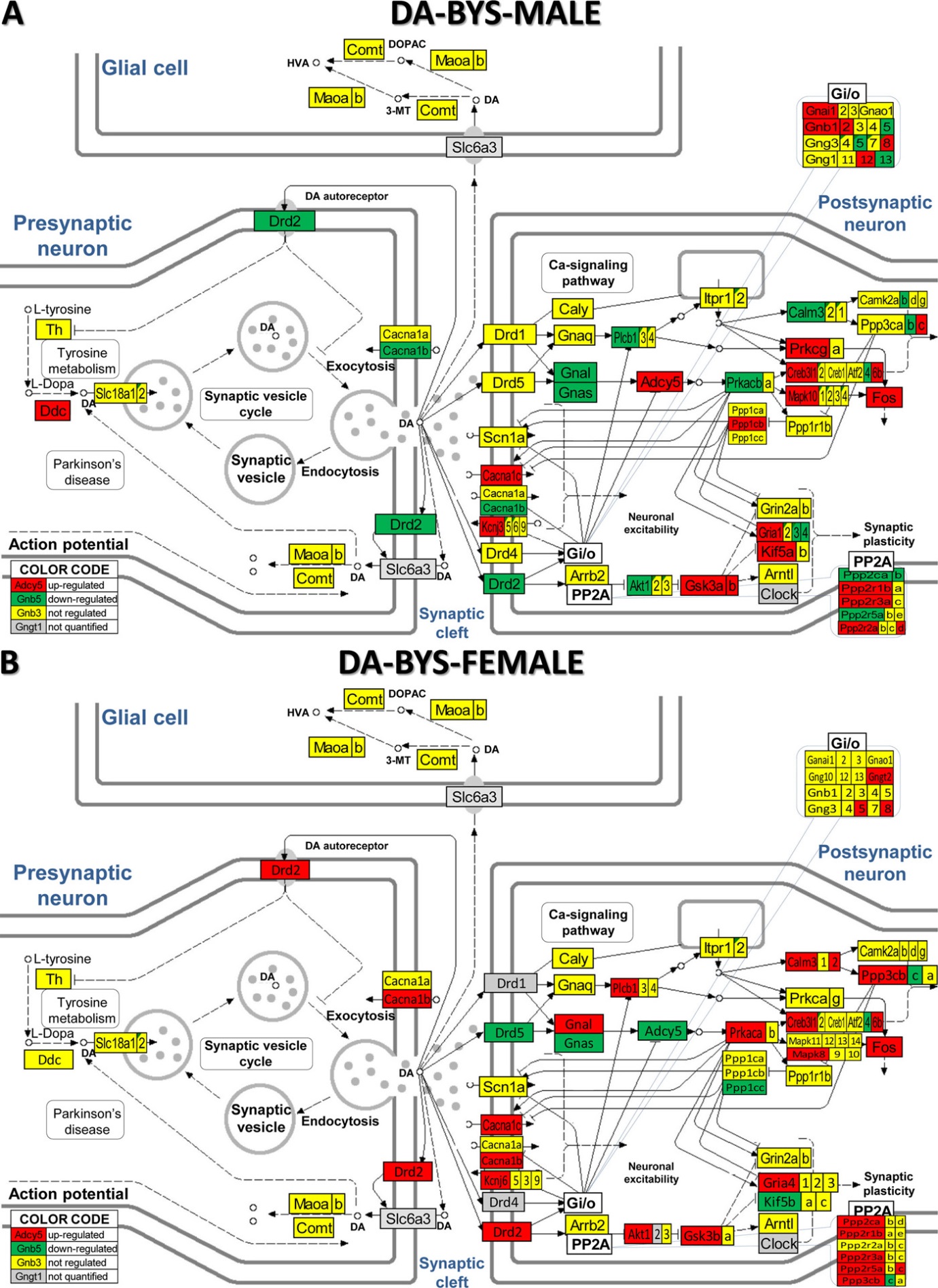
**

**KEGG (**www.kegg.jp/kegg/kegg1.html**) map of regulation of the dopaminergic synapse pathway in the arcuate nucleus of saline-treated betamethasone–primed male (A) and female (B) rats with NMDA-induced infantile spasms (BYS) compared to counterparts without spasms (BNS).**

**Supplementary Figure S3**

**
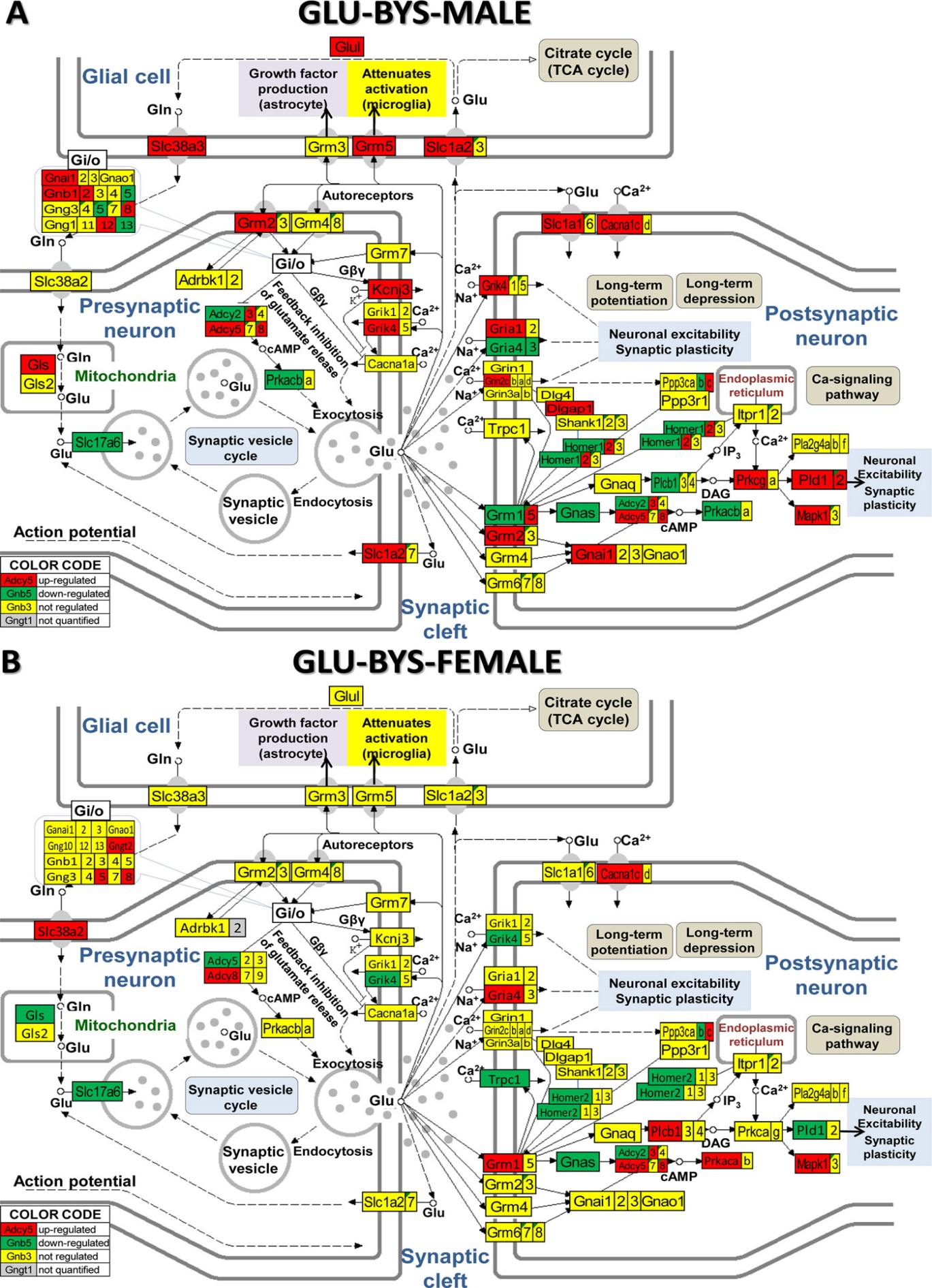
**

**KEGG (**www.kegg.jp/kegg/kegg1.html**) map of regulation of the glutamatergic synapse pathway in the arcuate nucleus of saline-treated betamethasone–primed male (A) and female (B) rats with NMDA-induced infantile spasms (BYS) compared to counterparts without spasms (BNS).**

**Supplementary Figure S4**

**
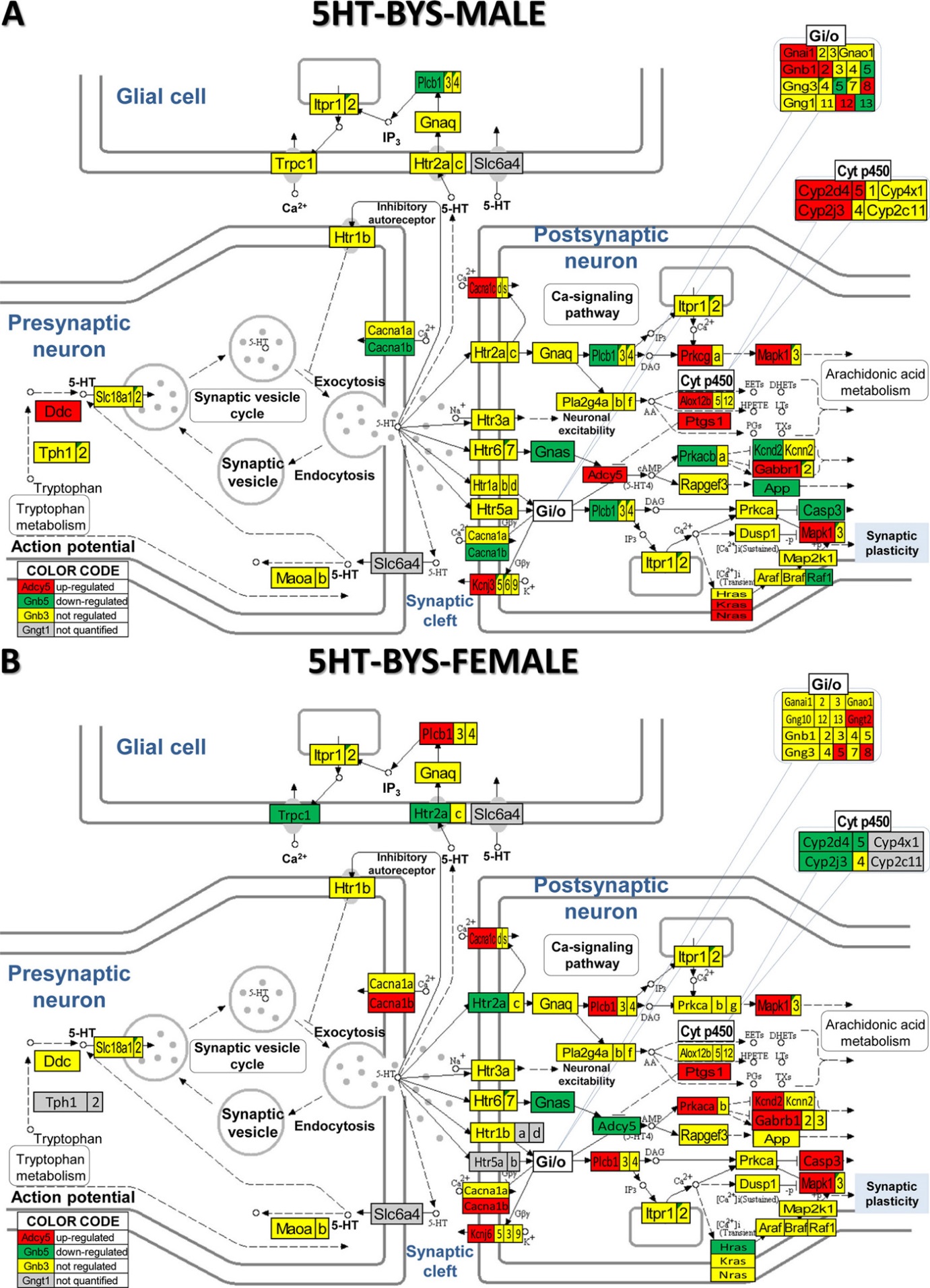
**

**KEGG (**www.kegg.jp/kegg/kegg1.html**) map of regulation of the serotonergic synapse pathway in the arcuate nucleus of saline-treated betamethasone–primed male (A) and female (B) rats with NMDA-induced infantile spasms (BYS) compared to counterparts without spasms (BNS).**

**
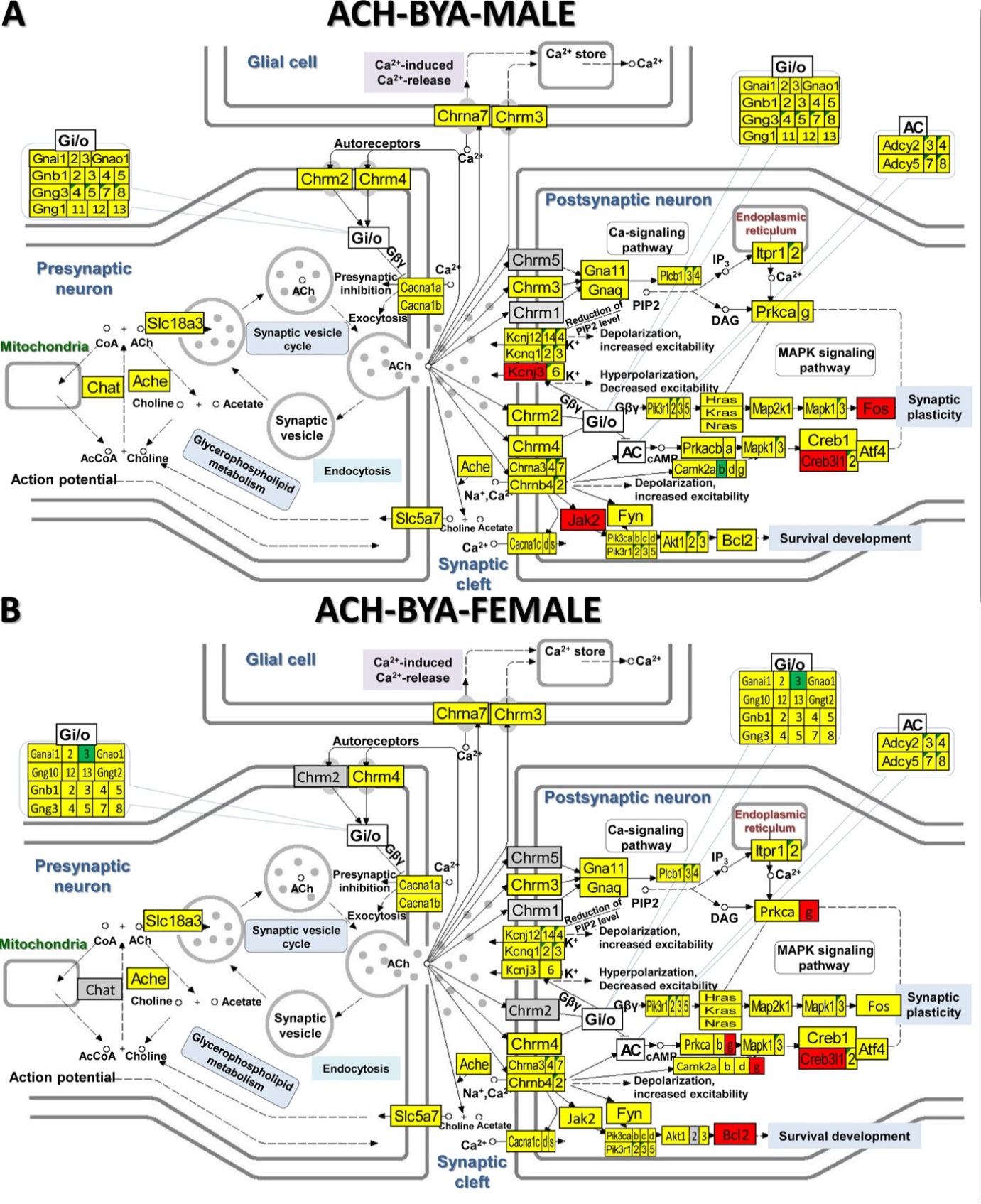
Supplementary Figure S5**

**KEGG (**www.kegg.jp/kegg/kegg1.html**) map of regulation of the cholinergic synapse pathway in the arcuate nucleus of ACTH-treated betamethasone–primed male (A) and female (B) rats with NMDA-induced infantile spasms (BYA) compared to counterparts without spasms (BNS).**

**Supplementary Figure S6**

**
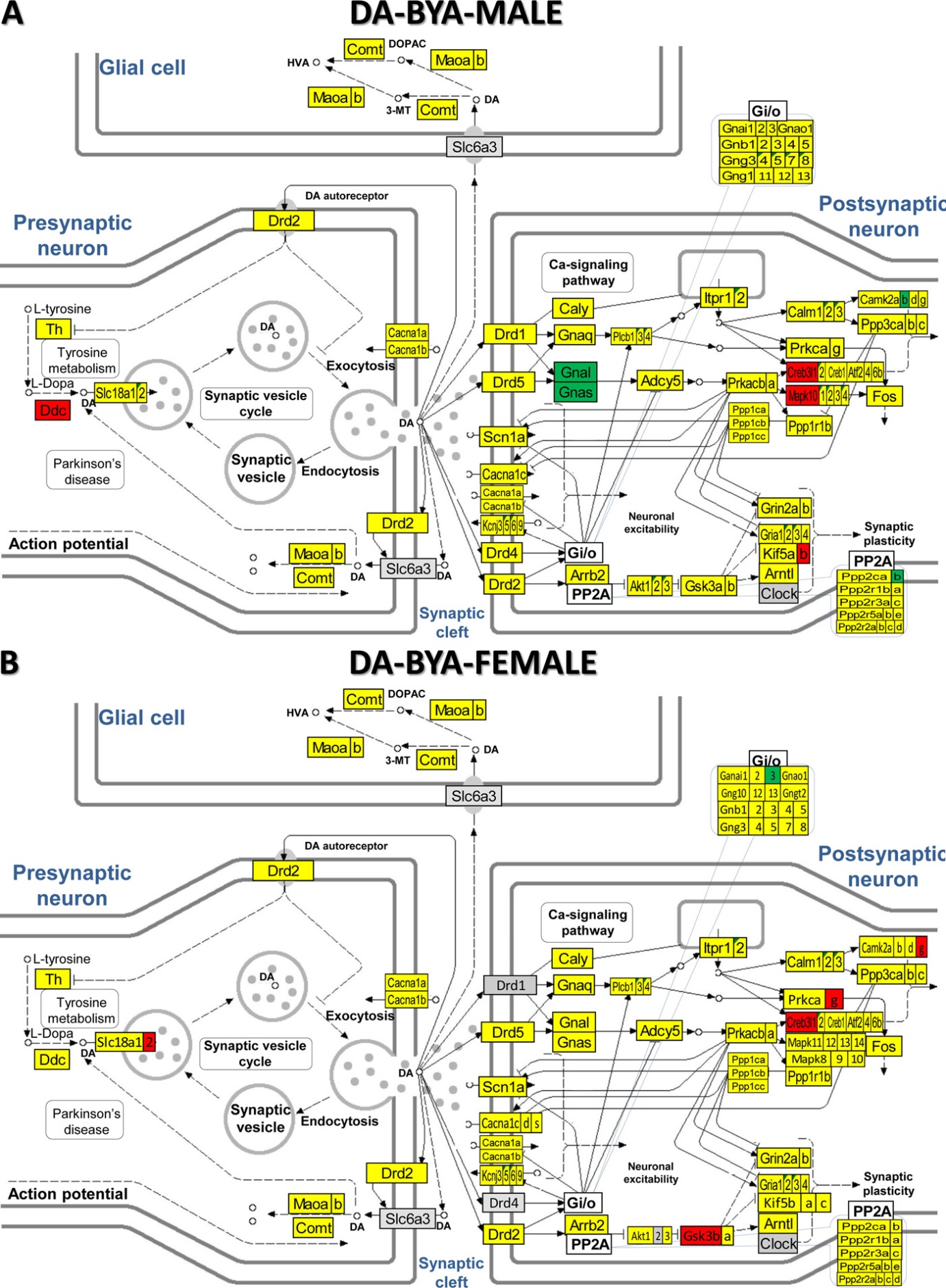
**

**KEGG (**www.kegg.jp/kegg/kegg1.html**) map of regulation of the dopaminergic synapse pathway in the arcuate nucleus of ACTH-treated betamethasone–primed male (A) and female (B) rats with NMDA-induced infantile spasms (BYA) compared to counterparts without spasms (BNS).**

**
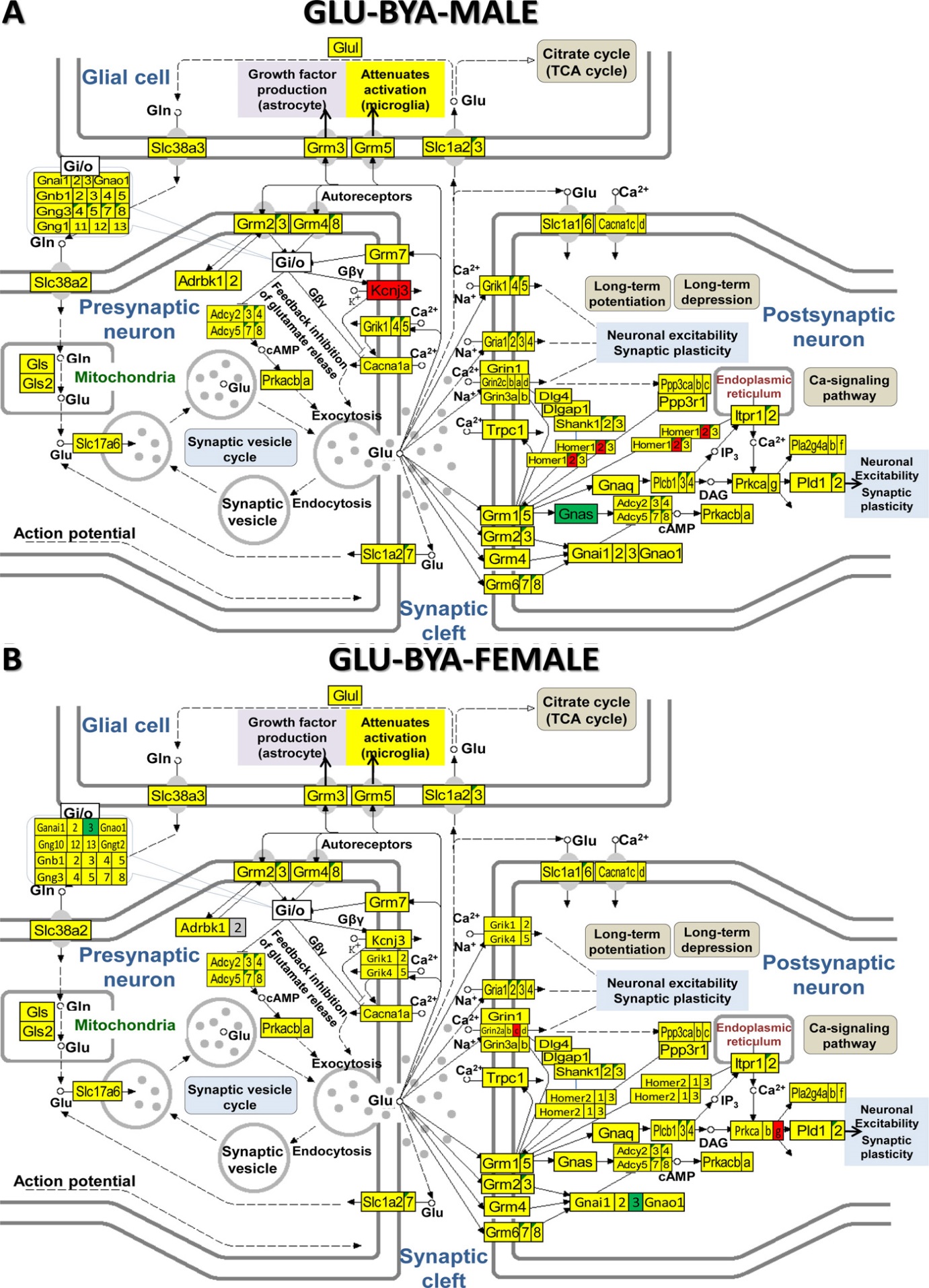
Supplementary Figure S7**

**KEGG (**www.kegg.jp/kegg/kegg1.html**) map of regulation of the gultaminergic synapse pathway in the arcuate nucleus of ACTH-treated betamethasone–primed male (A) and female (B) rats with NMDA-induced infantile spasms (BYA) compared to counterparts without spasms (BNS).**

**
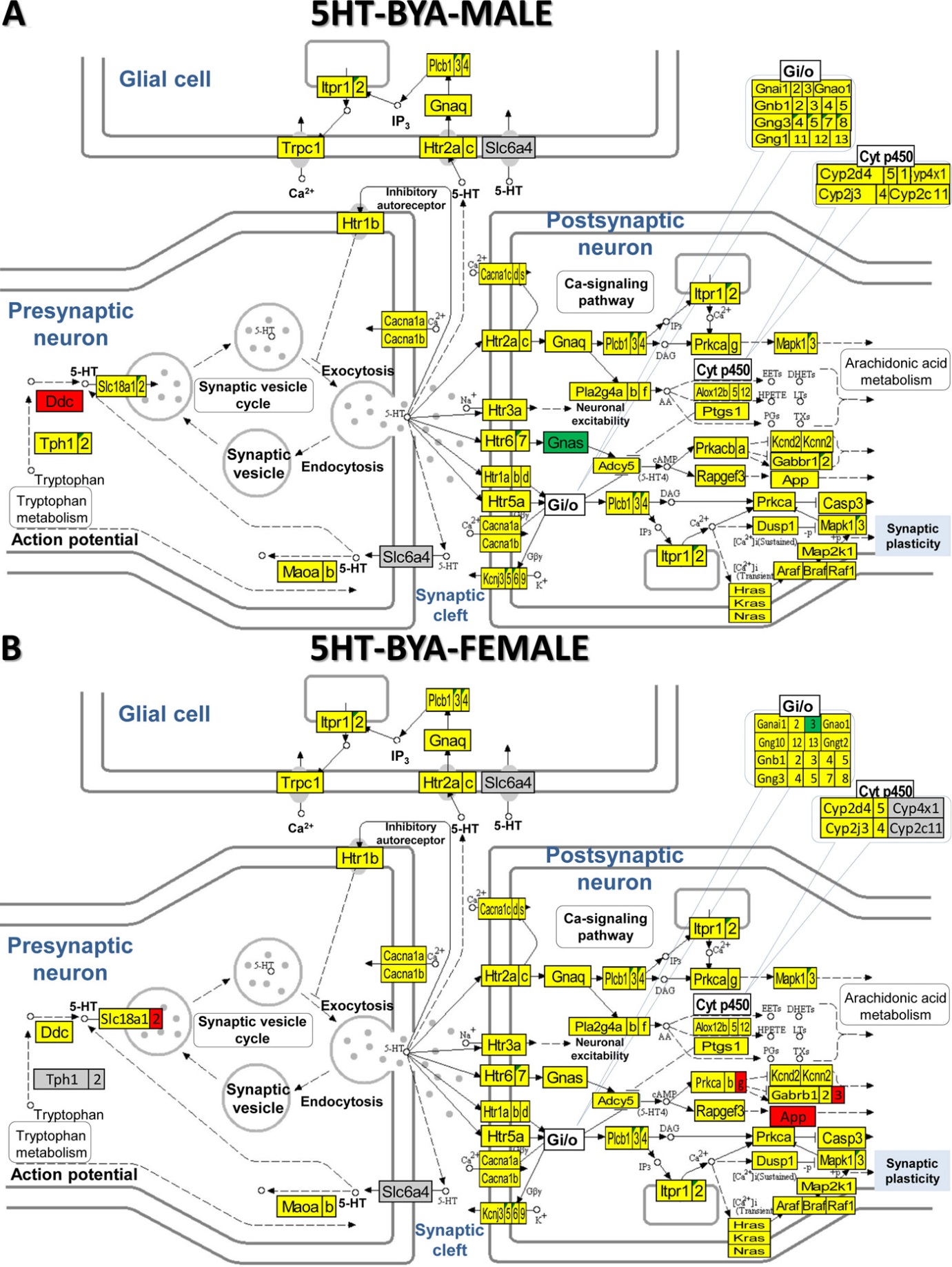
Supplementary Figure S8**

**KEGG (**www.kegg.jp/kegg/kegg1.html**) map of regulation of the dopaminergic synapse pathway in the arcuate nucleus of ACTH-treated betamethasone–primed male (A) and female (B) rats with NMDA-induced infantile spasms (BYA) compared to counterparts without spasms (BNS).**

**
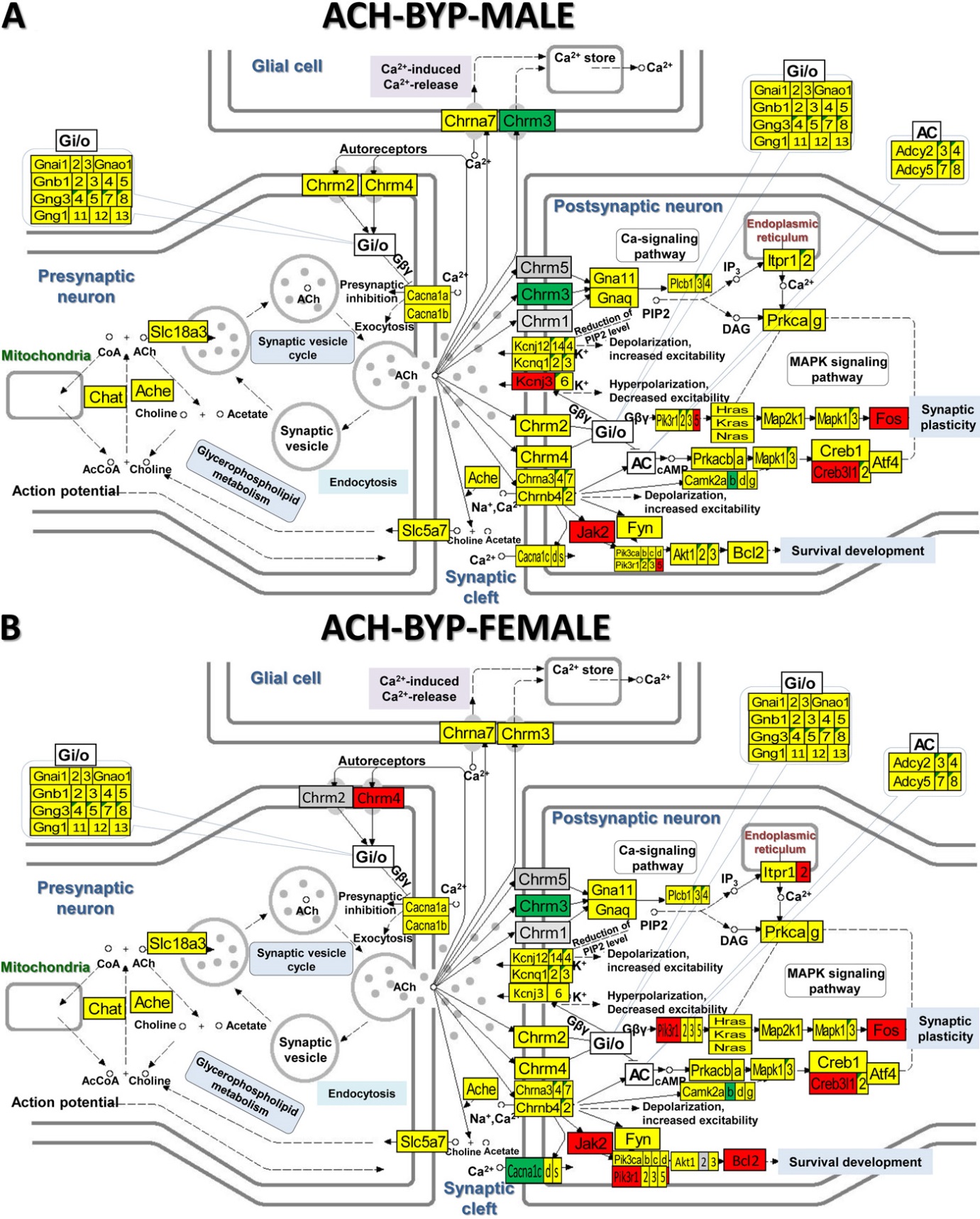
Supplementary Figure S9**

**KEGG (**www.kegg.jp/kegg/kegg1.html**) map of regulation of the cholinergic synapse pathway in the arcuate nucleus of PMX53-treated betamethasone–primed male (A) and female (B) rats with NMDA-induced infantile spasms (BYA) compared to counterparts without spasms (BNS).**

**
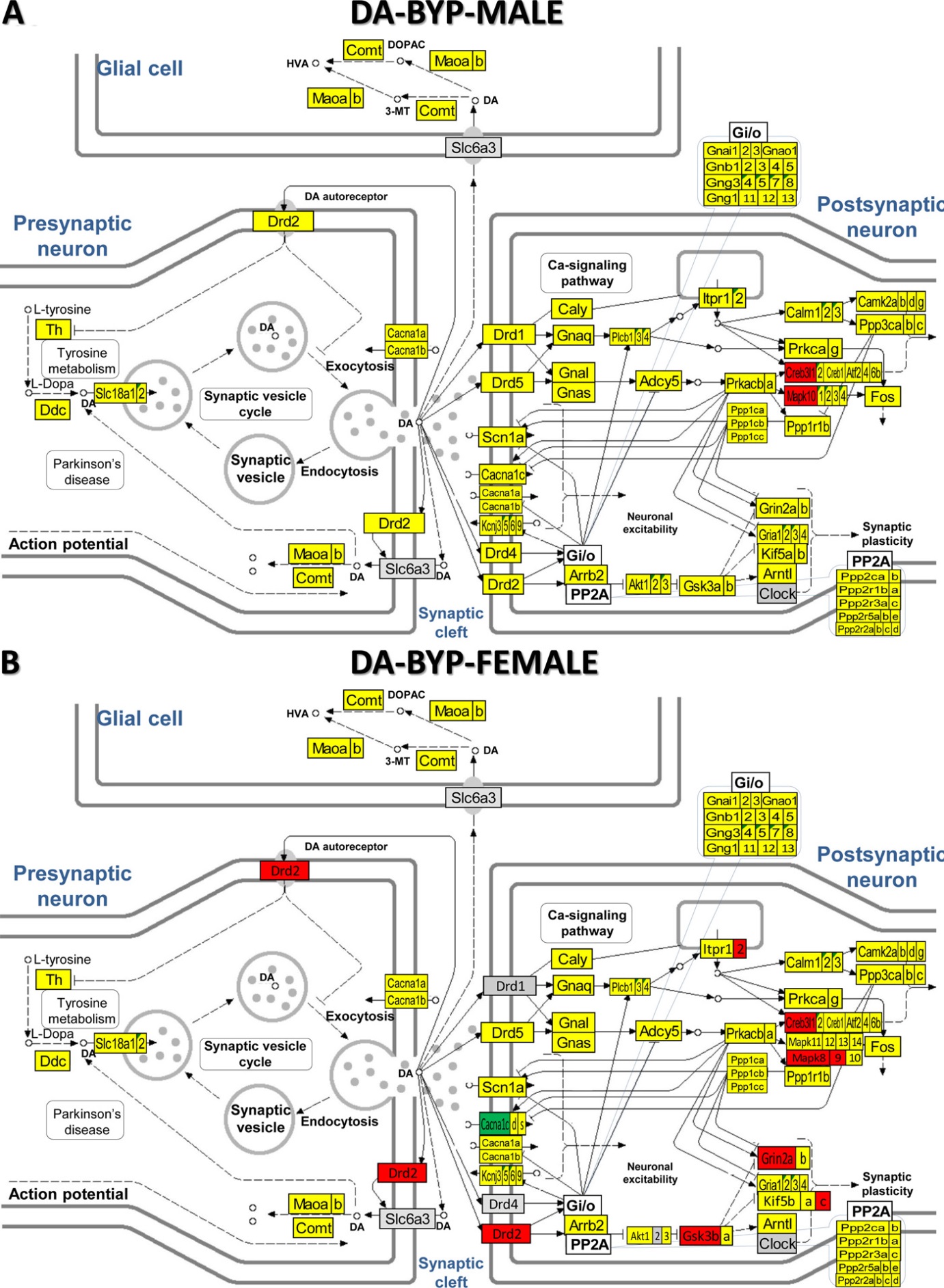
Supplementary Figure S10**

**KEGG (**www.kegg.jp/kegg/kegg1.html**) map of regulation of the dopaminergic synapse pathway in the arcuate nucleus of PMX53-treated betamethasone–primed male (A) and female (B) rats with NMDA-induced infantile spasms (BYA) compared to counterparts without spasms (BNS).**

**
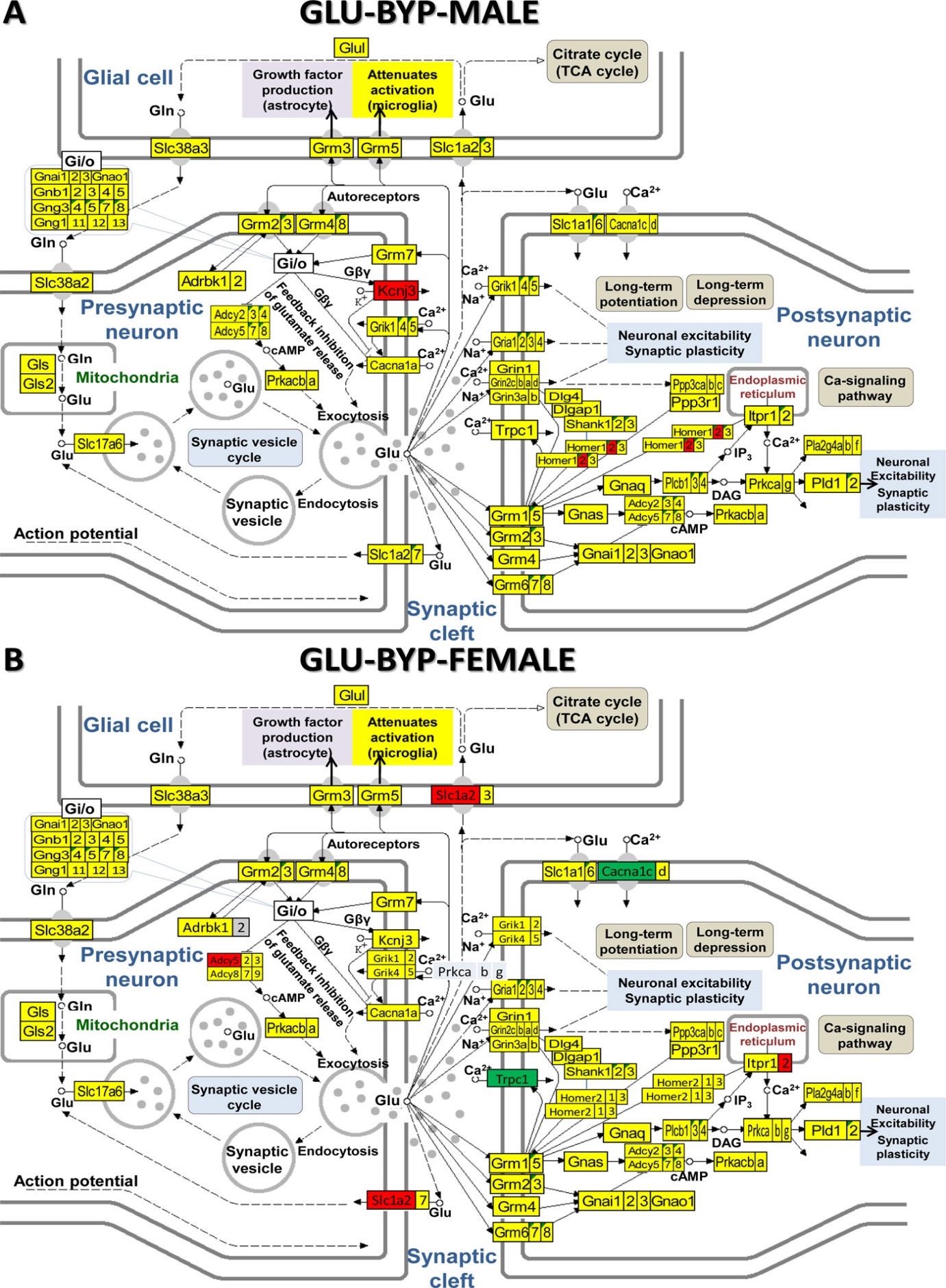
Supplementary Figure S11**

**KEGG (**www.kegg.jp/kegg/kegg1.html**) map of regulation of the glutaminergic synapse pathway in the arcuate nucleus of PMX53-treated betamethasone–primed male (A) and female (B) rats with NMDA-induced infantile spasms (BYA) compared to counterparts without spasms (BNS).**

**
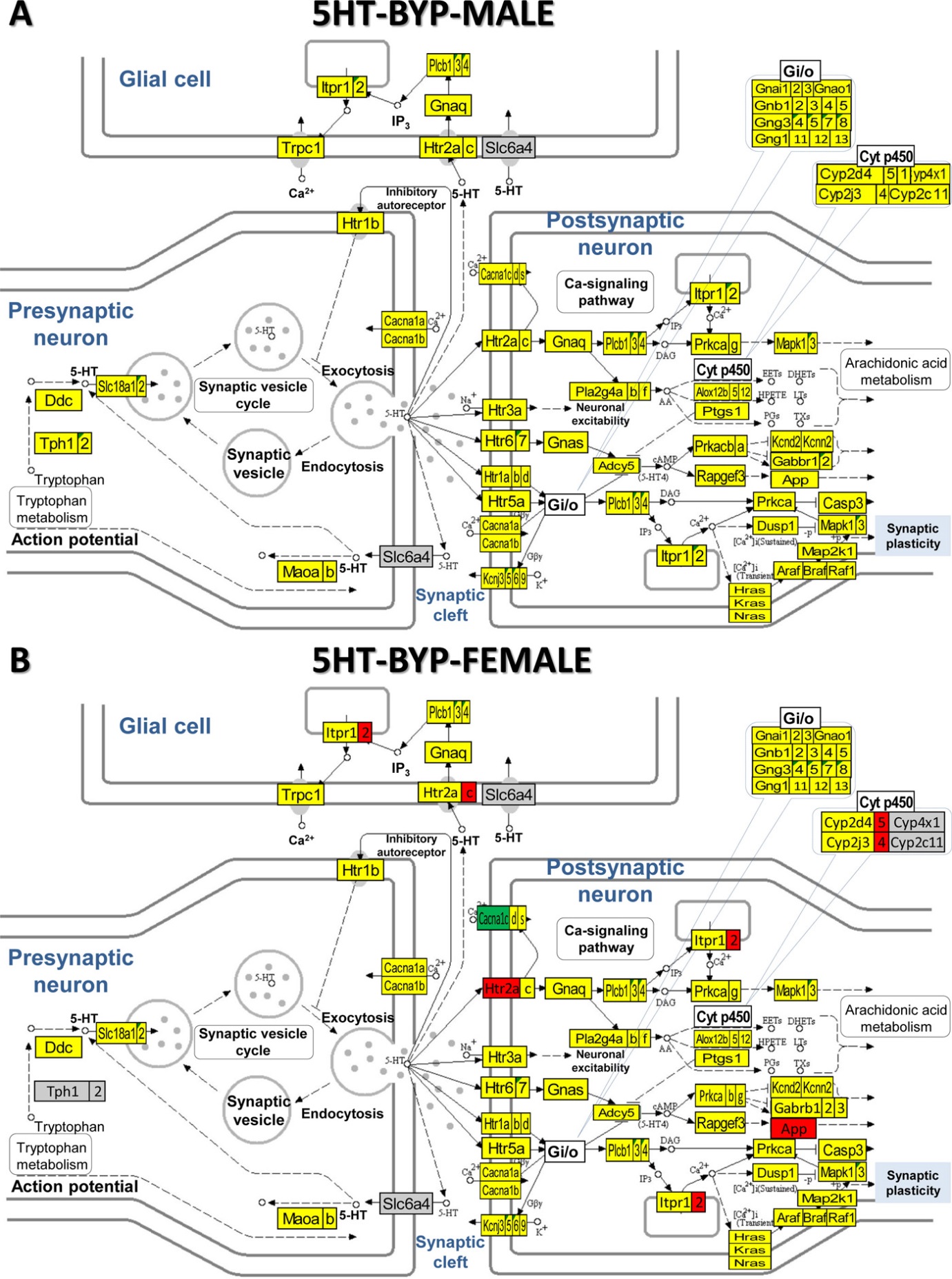
Supplementary Figure S12**

**KEGG (**www.kegg.jp/kegg/kegg1.html**) map of regulation of the serotonergic synapse pathway in the arcuate nucleus of PMX53-treated betamethasone–primed male (A) and female (B) rats with NMDA-induced infantile spasms (BYA) compared to counterparts without spasms (BNS).**
